# Band structures and scattering properties of the simplest one-dimensional ${\mathscr {P}}{\mathscr {T}}$-symmetric photonic crystal

**DOI:** 10.1038/s41598-022-20559-z

**Published:** 2022-09-27

**Authors:** Tiecheng Wang, Xiang Gou

**Affiliations:** 1 College of Physics and Electronic Engineering, Shanxi University, 030006 Taiyuan, China; 2 Institute of Theoretical Physics, Shanxi University, 030006 Taiyuan, China

**Keywords:** Photonic crystals, Photonic crystals

## Abstract

We elucidate the band structures and scattering properties of the simplest one-dimensional parity–time (${\mathscr {P}}{\mathscr {T}}$)-symmetric photonic crystal. Its unit cell comprises one gain layer and one balanced loss layer. Herein, the analytic expressions of the band structures and scattering properties are derived, and based on these relations, we reveal and explain the following phenomena: Exceptional point pairs appear from Brillouin boundaries at a nonzero non-Hermiticity. With an increase in non-Hermiticity, each of these pairs moves toward the Brillouin center, finally coalescing into a single point at the Brillouin center at a critical non-Hermiticity value. Near the exceptional point, singular scattering is observed and explained. This refers to the phenomenon whereby transmittances and reflectances for left and right incidences reach exceptionally large values simultaneously. Moreover, these are infinite at some discrete points at which poles and zeros of the scattering matrix are attained. In forbidden gaps, unidirectional weak visibility, where transmittances are zero, is disclosed and analyzed: specifically, the reflectance for incidence from one side is very large, whereas that for incidence from the other side is very small. In this phenomenon, the eigenstates of the scattering matrix are the incident waves from the left and right sides, and their eigenvalues are the corresponding reflectances. Our results are important as new functional optical devices can potentially be developed by utilizing these novel phenomena.

## Introduction

Considerable work has been devoted to ${\mathscr {P}}{\mathscr {T}}$-symmetric optical systems in recent years^1,2^. Such systems are characterized by a complex index of refraction with balanced gain and loss $n^*\left( -{\varvec{r}}\right) =n\left( {\varvec{r}}\right)$, and they exhibit notable regularities. The product of the two eigenvalues of the scattering matrix of such a system is one^3–5^. In a ${\mathscr {P}}{\mathscr {T}}$-exact phase, both eigenvalues are unimodular; therefore, the corresponding eigenstates exhibit no net amplification or dissipation. By contrast, in a ${\mathscr {P}}{\mathscr {T}}$-broken phase, the unimodularity condition cannot be satisfied, in which case one eigenstate corresponds to amplification and the other to dissipation^5^. In a special case, one eigenvalue is a zero corresponding to the scattering matrix, which can be used to realize a coherent perfect absorber^5–7^, and the other is the corresponding pole, which can be used to lase^6–10^. Furthermore, ${\mathscr {P}}{\mathscr {T}}$-symmetric photonic structures violate normal photon-flux conservation but obey generalized unitarity relations^11,12^. These relations are extended from one dimension to higher dimensions, wherein the interactions between multimode fields must be considered^13^.

Based on these regularities, many other extraordinary scattering phenomena have been detected and elucidated in ${\mathscr {P}}{\mathscr {T}}$-symmetric optical systems. Notably, anisotropic transmission resonances are supported, meaning that at some frequencies, there is unit transmission and zero reflection for a wave incident from a single side^11^. Unidirectional invisibility is a typical optical effect of ${\mathscr {P}}{\mathscr {T}}$-symmetric photonic structures^11,14–18^. The optical forces exerted on ${\mathscr {P}}{\mathscr {T}}$-symmetric photonic heterostructures under the normal incidence of a single and two counterpropagating plane waves have been studied^19^. In addition, the complex band structures of one-dimensional ${\mathscr {P}}{\mathscr {T}}$-symmetric photonic crystals (1DPTSPCs) have been calculated and analyzed; importantly, it has been found that with an increase in non-Hermiticity, two types of ${\mathscr {P}}{\mathscr {T}}$ phase diagrams are obtained^20,21^. The exceptional contours and complex band structures of two-dimensional ${\mathscr {P}}{\mathscr {T}}$-symmetric photonic crystals, whose non-Hermitian primitive cell corresponds to an integer multiple of the primitive cell of the underlying Hermitian system, have also been studied^22,23^. Besides the definitions of the ${\mathscr {P}}{\mathscr {T}}$-exact phase and ${\mathscr {P}}{\mathscr {T}}$-broken phase based on the eigen equations of the scattering matrixes, there are another definitions based on complex band structures^20–23^. In our previous work, we clarified these two defining criteria and suggested that the definitions based on complex band structures are preferable^24^.

Herein, we study the transmittances and reflectances of the simplest one-dimensional ${\mathscr {P}}{\mathscr {T}}$-symmetric photonic crystal (SOPTPC) comprehensively and thoroughly. Further, we present theoretical expressions obtained using the simplicity of this model. The rest of this paper is organized as follows: In section “Model and structure”, the analytic expressions of the transfer matrix and band structure are derived; further, these are used to elucidate the band structures for different non-Hermiticities. In section “Scattering properties”, we disclose and discuss the unidirectional weak visibility and singular scattering phenomena in our model. We conclude the paper with a summary in section “Conclusion”.

## Model and structure

**Figure 1:**
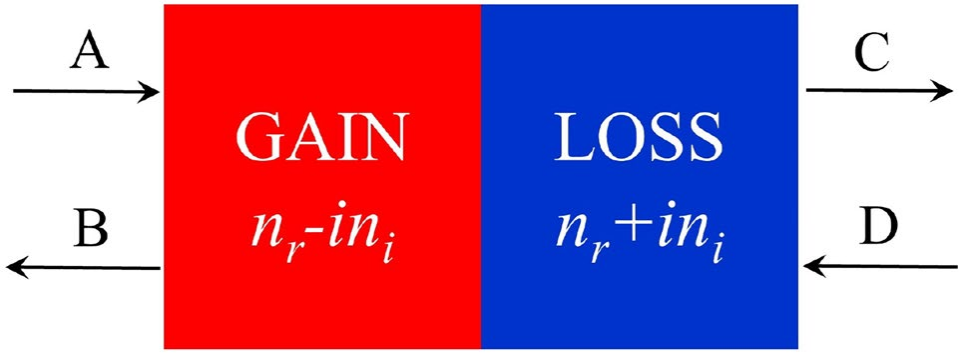
Schematic of the unit cell of the SOPTPC, which comprises two layers with refractive indices $n^*\equiv n_r-in_i$ and $n\equiv n_r+in_i$; the thicknesses of the two layers in the primitive cell are equal, $d=0.5\Lambda$.

Here, the object of study is the SOPTPC system, in which each unit cell consists of two alternating layers, as shown in Fig. 1. Their refractive indices are represented by $n^*\equiv n_r-in_i$ and $n\equiv n_r+in_i$; further, their thicknesses are equal and are denoted by $d=0.5\Lambda$, where $\Lambda$ is the thickness of a unit cell. In this study, as shown in Fig. 1, we only consider the simplest case, wherein the electromagnetic wave in the model propagates normally. *A* (*C*) and *B* (*D*) represent the amplitudes of the incident and reflected waves on the left (right) side of a unit cell, respectively. By using the transfer matrix method and Bloch’s theorem, we can obtain the Bloch wave vector and Bloch state from the eigen-equation of the transfer matrix of a unit cell:

1 $$\begin{aligned} \left( \begin{array}{cc} a^* &{} ic \\ -ib &{} a \end{array} \right) \left( \begin{array}{c} A \\ B \end{array} \right) =e^{iK\Lambda }\left( \begin{array}{c} A \\ B \end{array} \right) . \end{aligned}$$

Here, *K* denotes the Bloch wave vector, and the parameters corresponding to the transfer matrix are given by

2a $$\begin{aligned} c= & {} \frac{1}{n^2_r+n^2_i}\left[ -n_rn_i{\cos \left( 2k_rd\right) }+n_rn_i{\cosh \left( 2k_id\right) }\right. \nonumber \\&\left. +{n_r\left( n^2_r+n^2_i -1\right) }{\sin \left( 2k_rd\right) }/2-{n_i\left( n^2_r+n^2_i+1\right) }{\sinh \left( 2k_id\right) }/2\right] , \end{aligned}$$

2b $$\begin{aligned} b= & {} \frac{1}{n^2_r+n^2_i}\left[ n_rn_i{\cos \left( 2k_rd\right) }-n_rn_i{\cosh \left( 2k_id\right) }\right. \nonumber \\&\left. +{n_r\left( n^2_r+n^2_i-1\right) }{\sin \left( 2k_rd\right) }/2-{n_i\left( n^2_r+n^2_i+1\right) }{\sinh \left( 2k_id\right) }/2\right] , \end{aligned}$$

2c $$\begin{aligned} a= & {} \frac{1}{n^2_r+n^2_i}\left[ n^2_r{\cos \left( 2k_rd\right) }\right. \nonumber \\&\left. +n^2_i{\cosh \left( 2k_id\right) }-i{n_r\left( n^2_r+n^2_i+1\right) }{\sin \left( 2k_rd\right) }/2+i{n_i\left( n^2_r+n^2_i-1\right) }{\sinh \left( 2k_id\right) }/2\right] , \end{aligned}$$

Here, we assume that each unit cell is surrounded by infinitely thin air-films on both sides. $\omega$ and $c_0$ represent the angular frequency and velocity of plane waves in vacuum, and *k* is the wave vector in the lossy layer, $k\equiv k_r+ik_i=\omega n/c_0$. Further, it can be clearly observed that *a* is complex and *b* and *c* are real, and they satisfy $|a|^2=bc+1$. This relation also holds for other ${\mathscr {P}}{\mathscr {T}}$-symmetric photonic heterostructures^11,12^.

From Eq. (2), we can derive the analytic expression of band structures, which presents the relation between the Bloch wave vector *K* and frequency $\omega$

3 $$\begin{aligned} \cos \left( K\Lambda \right) =\frac{1}{n^2_r+n^2_i}\left[ n^2_r{\mathrm {cos} \left( 2k_rd\right) \ }+n^2_i{\cosh \left( 2k_id\right) \ }\right] . \end{aligned}$$

**Figure 2:**
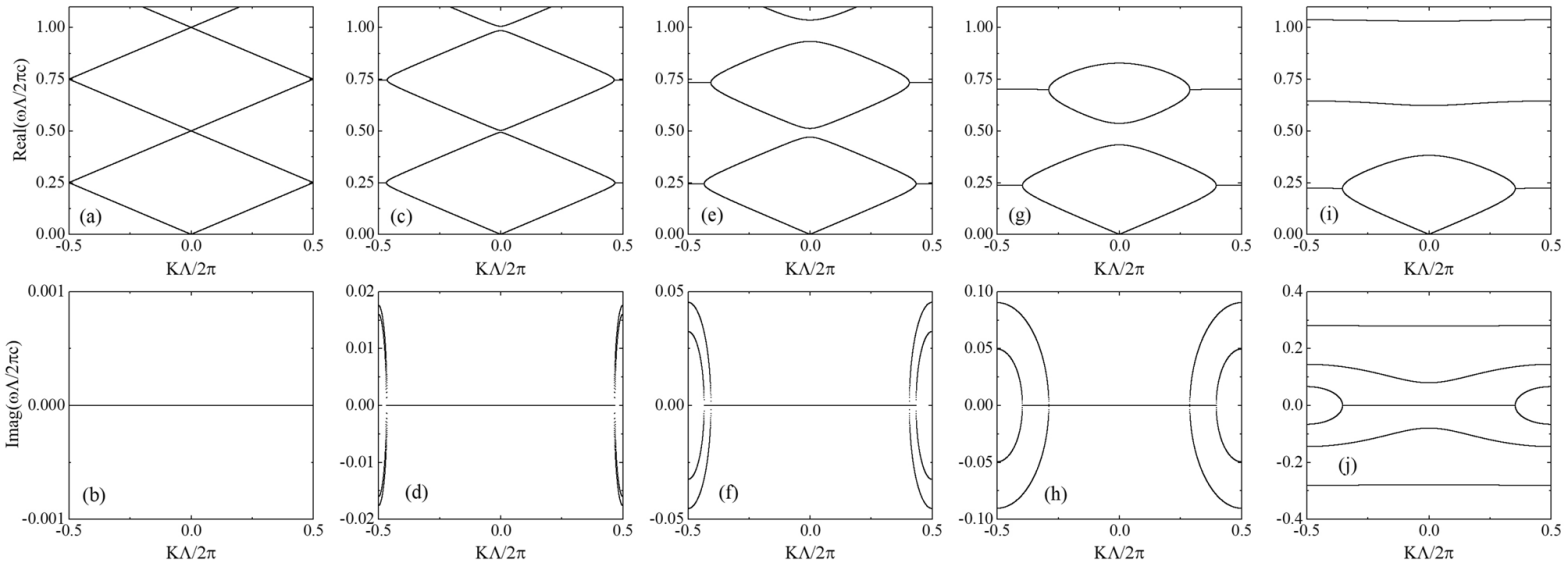
Complex band structures of the SOPTPC at $n_i=0$ (**a**) and (**b**), $n_i=0.2$ (**c**) and (**d**), $n_i=0.4$ (**e**) and (**f**), $n_i=0.6$ (**g**) and (**h**), and $n_i=0.8$ (**i**) and (**j**). The five panels at the top show the real parts of the reduced frequencies, and the five panels in the bottom denote the corresponding imaginary parts of the reduced frequencies. The real parts of the refractive indices are fixed at $n_r=2.0$.

Notably, because the right side of this equation is real, the Bloch wave vector can be real or complex, with the form $2m\pi /\Lambda +iK_i$, where *m* is an integer and $K_i$ is the imaginary part of the Bloch wave vector, and $\omega$ may be complex. Note that $-K$ and $\omega$ also satisfy this relation. If $\omega$ is real, the system is in the ${\mathscr {P}}{\mathscr {T}}$-exact phase, and if $\omega$ is complex, *K* and $\omega ^*$ also satisfy this relation, which corresponds to the ${\mathscr {P}}{\mathscr {T}}$-broken phase. An exceptional point is located at the boundary between these two phases. At the exceptional point, the condition $dK/d\omega =0$ holds for real frequencies and real Bloch wave vectors in the interval $(0, \pi )$, which can be understood easily from band structure diagrams such as the one presented in the Fig. 2. Moreover, by substituting Eq. (3) into this condition, we can obtain the position of the exceptional point in SOPTPC theoretically, by solving the following transcendental equation:

4 $$\begin{aligned} n_r^3\sin \left( \omega n_r\Lambda /c_0\right) =n_i^3\sinh \left( \omega n_i\Lambda /c_0\right) . \end{aligned}$$

Any band structure of the SOPTPC can be obtained by using Eq. (3); when we calculate these by using a computer, real frequencies are often scanned, and the aforenoted equation is used to solve for the Bloch wave vector. It is difficult to handle the cases wherein the frequency is complex by using that equation directly. This complex band structure can be calculated by the method of superposition of Bloch states in the absence of gain and loss^20^. This method can be applied to the SOPTPC as follows: the Bloch state of the underlying Hermitian system without gain and loss can be described as $E^0_{mK}=u^0_{mK}\left( x\right) e^{iKx}$, with the corresponding frequency ${\omega }^0_{mK}$; further, the Bloch state of the corresponding ${\mathscr {P}}{\mathscr {T}}$-symmetric system $E_{mK}=u_{mK}\left( x\right) e^{iKx}$ can be expressed as a superposition of the Bloch states in absence of gain and loss $u_{mK}\left( x\right) =\sum ^{\infty }_{m^\prime =1}{{\eta }_{m,m^\prime K}u^0_{m^\prime K}\left( x\right) }$. Truncation is performed in the practical calculation of this summation ($m^\prime =1, 2 \ldots M$). After substituting this expansion into the Helmholtz equation, the eigen equation of the effective Hamiltonian can be derived:

5 $$\begin{aligned} H_K{\eta }_{mK}={\left( \frac{{\omega }_{mK}}{c_0}\right) }^2{\eta }_{mK}. \end{aligned}$$

Here, $H_K=H^{-1}_{1K}H_{2K}$; further, ${\left( H_1\right) }_{m^\prime m}={\left( {\overline{\varepsilon }}_{rK}\right) }_{m^\prime m}+i{\left( {\overline{\varepsilon }}_{iK}\right) }_{m^\prime m}$ and ${\left( H_2\right) }_{m^\prime m}={\left( {{\omega }_{mK}}/{c_0}\right) }^2{\left( {\overline{\varepsilon }}_{rK}\right) }_{m^\prime m}$ are associated with ${\left( {\overline{\varepsilon }}_{r,iK}\right) }_{m^\prime m}=\int {dx u^{0*}_{m^\prime K}\left( x\right) {\varepsilon }_{r,i}\left( x\right) u^0_{mK}\left( x\right) }$. Here, the $M\times M$ matrix $H_K$ can be regarded as the effective Hamiltonian for the ${\mathscr {P}}{\mathscr {T}}$-symmetric system, and ${\eta }_{mK}={\left( {\eta }_{m,1K},\ {\eta }_{m,2K}\cdots {\eta }_{m,MK}\right) }^T$ is the eigenvector; the corresponding eigen frequency is ${\omega }_{mK}$. For our model, ${\omega }^0_{mK}=\pm Kc_0/{n_r}+2\pi c_0 m/\left( {n_r}\Lambda \right)$ and $u^0_{mK}\left( x\right) =e^{i\left( K\pm 2m\pi /\Lambda \right) x}$. Moreover, the diagonal elements of matrix ${\overline{\varepsilon }}_{rK}$ are equal to ${\varepsilon }_r\Lambda$, and the non-diagonal elements are equal to zero, whereas the diagonal elements of matrix ${\overline{\varepsilon }}_{iK}$ are equal to zero and the non-diagonal elements ${\left( {\overline{\varepsilon }}_{iK}\right) }_{m^\prime m}$ are $\frac{\Lambda }{i\left( m-m^\prime \right) \pi }{\varepsilon }_i\left[ 1-{\left( -1\right) }^{\left( m-m^\prime \right) }\right]$.

In Fig. 2, we plot the complex band structures for different values of non-Hermiticities $n_i$; the real part is fixed at $n_r=2.0$^25^, and the 30 lowest bands in absence of gain and loss are considered to expand the Bloch waves in the SOPTPC. The band structure in the absence of gain and loss is shown in Fig. 2a and b. The analytic expression can be derived from Eq. (3), $\frac{{\omega }_{mK} \Lambda }{2\pi c_0}=\pm \frac{K\Lambda }{2\pi n_r}+\frac{m}{n_r}$, where $m=0, 1, 2\ldots$. In this case, the system is homogeneous, and we can observe that there is no forbidden gap. As the imaginary part of the refractive index increases, e.g., as shown in Fig. 2c and d at $n_i=0.2$, exceptional point pairs emerge from the Brillouin boundaries, the bands open, and the corresponding bandgaps appear at the Brillouin center. From Eq. (3), it can be proved, as follows, that exceptional points emerge for a nonzero non-Hermiticity $n_i$ regardless of the smallness of $n_i$. At Brillouin boundaries $K\Lambda =\pm \pi$, if the frequency is real, then $-1<-n^2_r{\cos \left( 2k_0n_rd\right) /\left( n^2_r+n^2_i\right) }<1$ and ${n^2_i}{\cosh \left( k_i\Lambda \right) }/\left( n^2_r+n^2_i\right) >0$; therefore, Eq. (3) cannot be satisfied, and the frequency must be complex. Exceptional points always appear in pairs owing to mirror symmetry in *K* space, which is discussed in a previous theoretical analysis^1,2^. It can also be proved from Eq. (3) that forbidden gaps are located at Brillouin centers for a nonzero $n_i$: At zero non-Hermiticity, bands touch at the Brillouin center, and the frequencies corresponding to the points where the bands touch are located at $\frac{\omega _{mK}\Lambda }{2\pi c_0}=\frac{m}{2}$; further, at a nonzero non-Hermiticity, if the previous band-touching points satisfy the dispersion relation given by Eq. (3), then $1=\left[ {n^2_r}+{n^2_i}{\cosh \left( k_i\Lambda \right) \ }\right] /(n^2_r+n^2_i)$; therefore, ${\cosh \left( k_i\Lambda \right) \ }=1$; however, $k_i\Lambda$ cannot be zero under our assumption. Consequently, forbidden gaps appear at these frequencies.

As the non-Hermiticity $n_i$ is increased continuously, the forbidden gaps broaden gradually, and exceptional point pairs move away from the Brillouin boundaries to the Brillouin center; at some critical values, these exceptional point pairs coalesce at the Brillouin center. Subsequently, the corresponding conduction bands completely evolve into complex bands, wherein the eigen frequency corresponding to any Bloch wave vector is complex. The critical values can be solved theoretically; specifically, they can be determined by Eqs. (4) and (3) at $K=0$. After a lengthy albeit straightforward calculation, these critical points can be determined based on the following expression:

6 $$\begin{aligned} \cosh \left( \omega n_i\Lambda /c_0\right) =2n_r^2/n_i^2-1. \end{aligned}$$

**Figure 3:**
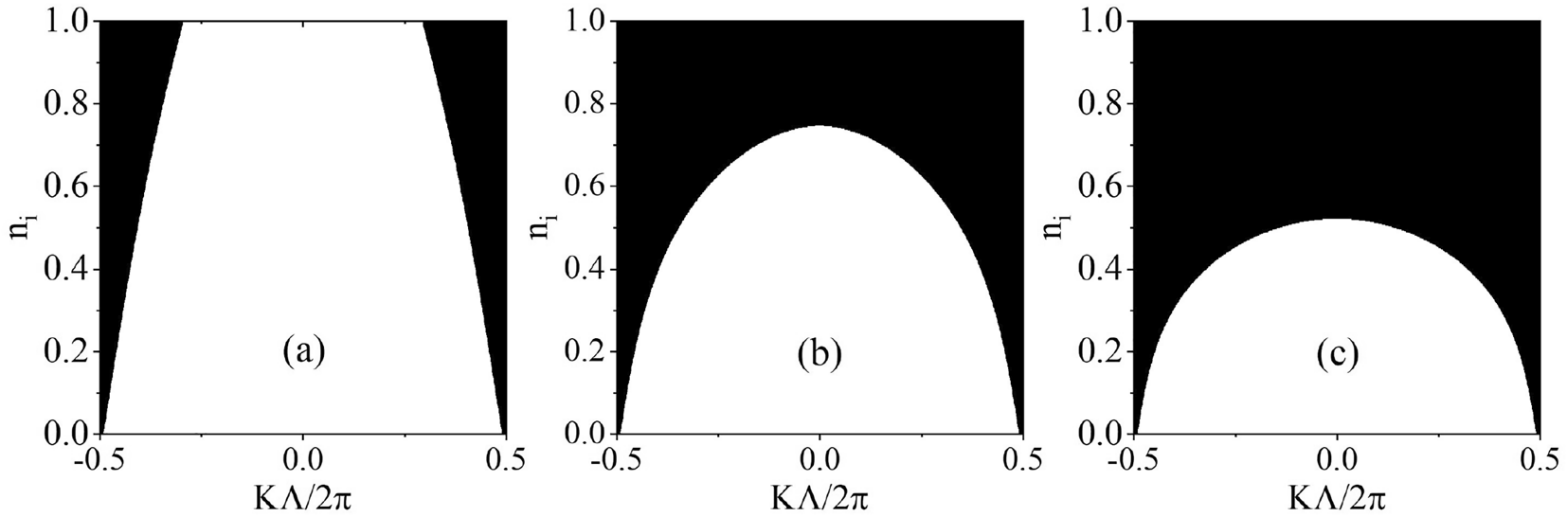
Phase diagrams in ($K\Lambda /2\pi$, $n_i$) space below the first (**a**), second (**b**), and third (**c**) forbidden gaps. The shaded parts correspond to the ${\mathscr {P}}{\mathscr {T}}$-broken phase and the white parts to the ${\mathscr {P}}{\mathscr {T}}$-exact phase.

In addition, for any fixed non-Hermiticity $n_i$, we find that the larger the frequency of the exceptional point pair, the closer the pair gets to the Brillouin center. This phenomenon can be also explained theoretically: if the frequency is large, then ${\cosh \left( k_i\Lambda \right) }$ is large according to Eq. (4); consequently, the minimum of ${\cos \left( K\Lambda \right) \ }$ becomes large. Hence, as $n_i$ increases, the conduction bands with larger frequencies evolve to complete the complex band more rapidly, which can be also observed in the Fig. 3.

From the complex band structures, we can identify the ${\mathscr {P}}{\mathscr {T}}$-broken phase from the ${\mathscr {P}}{\mathscr {T}}$-exact phase. In Fig. 3a–c, we plot the phase diagrams in (${K\Lambda }/{2\pi }$, $n_i$) space for the bands under the first, second, and third band gaps, respectively. The shaded areas represent ${\mathscr {P}}{\mathscr {T}}$-broken phases, and the white areas denote ${\mathscr {P}}{\mathscr {T}}$-exact phases. The boundaries between the two phases are the trajectories of exceptional points. As non-Hermiticity is increased, exceptional points appear in pairs and move away from the Brillouin boundaries to the Brillouin center. Notably, at some critical values, they coalesce at the Brillouin center; concurrently, the corresponding bands evolve into two complete complex conjugate bands.

## Scattering properties

In this section, we analyze the scattering properties of SOPTPC with *N* unit cells, in which case the frequency $\omega$ is considered to be real. As demonstrated by^24,26,27^, the transmittance $T_N \equiv |t_N|^2$ for the incidences from both sides, the reflectance $R_{NL} \equiv |r_{NL}|^2$ for the incidence from the left side, and the reflectance $R_{NR} \equiv |r_{NR}|^2$ for the incidence from the right side can be expressed as

7a $$\begin{aligned} T_N= & {} \frac{1}{bcU_N^2+1}, \end{aligned}$$

7b $$\begin{aligned} R_{NL}= & {} \frac{b^2}{bc+1/U_N^2}, \end{aligned}$$

7c $$\begin{aligned} R_{NR}= & {} \frac{c^2}{bc+1/U_N^2}, \end{aligned}$$

The above expressions help prove that the generalized conservation law for any ${\mathscr {P}}{\mathscr {T}}$-symmetric system holds true for $\left| T_N-1\right| =\sqrt{R_{NL}R_{NR}}$. According to Eq. (1), $bc=1/\left( n^2_r+n^2_i\right) ^2\left( u^2-v^2\right)$, where $u={n_r\left( n^2_r+n^2_i-1\right) }{\sin \left( 2k_rd\right) }/2-{n_i\left( n^2_r+n^2_i+1\right) }{\sinh \left( 2k_id\right) }/2$ and $v=n_rn_i{\cos \left( 2k_rd\right) }-n_rn_i{\cosh \left( 2k_id\right) }$.

**Figure 4:**
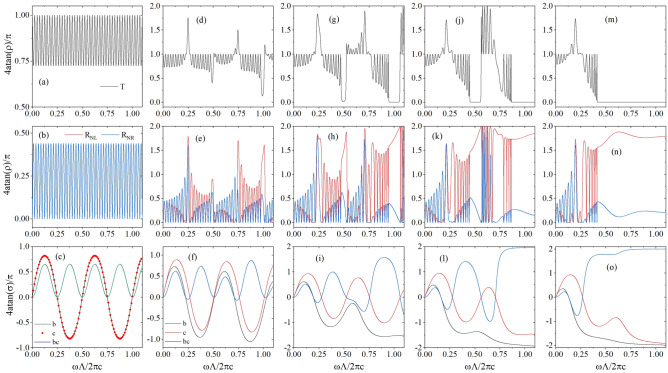
Transmittance, reflectances, and corresponding parameters in the transfer matrix of the simplest $${\mathscr {P}}{\mathscr {T}}$$-symmetric photonic crystal with $$N=10$$ periods at $$n_i=0$$ (**a**)–(**c**), $$ n_i=0.2$$ (**d**)–(**f**), $$n_i=0.4$$ (**g**)–(**i**), at $$n_i=0.6$$ (**j**)–(**l**), at $$n_i=0.8$$ (**m**)–(**o**). $$T_N$$ is denoted by the black lines in the top five panels. $$R_{NL}$$ and $${R}_{NR}$$ are represented by the red and blue lines in the middle five panels. The parameters *b*, *c*, and *bc* in the transfer matrix of unit cell are shown by black, red, and blue lines in the bottom five panels. The real parts of refractive indices are fixed at $$n_r=2.0$$.

Figure 4 shows the transmittances and reflectances from both sides at different values of non-Hermiticity $n_i$. To analyse these properties theoretically using Eq. (7), we plot the parameters *b*, *c*, and *bc* in the bottom panels. The number of unit cells here is set as $N=10$. The values ($T_N$, $R_{NL}$ and $R_{NR}$) are denoted by $\rho$ and are extremely large in some frequency regions. To observe their variations, the new ordinate $y=4{{\tan }^{-\mathrm {1}}\rho }/\pi$ is set as follows: If $\rho =1$, $y=1$, and if $\rho$ approaches $\pm \infty$, *y* approaches $\pm 2$. By combining Figs. 2 and 4, the transmittance at the band gap is zero; thus, scattering properties are observed to be closely related to band structures. In the absence of gain and loss $n_i=0$, $b=c$, the system is in a ${\mathscr {P}}{\mathscr {T}}$-exact phase, and all bands overlap each other at Brillouin boundaries and the Brillouin center; further, $R_{NL}=R_{NR}$ is valid here, which is demonstrated using Eqs. (7b) and (7c). For every conduction band, $K\Lambda$ varies from -$\pi$ to $\pi$; in this range, $N-1=9$ zero points exist for $K\Lambda =m\pi /N$ $\left( m=1,\ 2,\cdots N-1\right)$ for $U_N^2$. Therefore, at these frequencies, the transmittances arrive at the maximum 1, which can be understood from Eq. (7a). Simultaneously, the reflectances from both sides reach the minimum $R_{NL}=R_{NR}=0$, which can be understood from Eqs. (7b) and (7c).

When the non-Hermiticity $n_i$ is increased, the system is no longer homogeneous, and band gaps are formed due to interference of waves scattered by unit cells, and forbidden gaps between conduction bands become increasingly large. In forbidden gaps, transmittance is zero $T_N=0$, and the gap between $R_{NL}$ and $R_{NR}$ are extremely large. This is demonstrated using Eq. (7), $K{\Lambda }=2m\pi +iK_i{\Lambda }$ in forbidden gaps, then $U_N={\sinh NK_i\Lambda }/{\sinh K_i\Lambda }$. For a large number of unit cells *N*, the numerator of $U_N$ is considerably larger than its denominator due to the property of the hyperbolic function $\sinh (x)$, and therefore, $U_N^2$ takes an extremely large value. Consequently, the transmittance in Eq. (7a) reaches zero. Moreover, because $U_N^2$ is extremely large, $R_{NL}=b/c$ and $R_{NR}=c/b$; then, from the bottom five panels, in forbidden gaps, one reflectance is small and the other reflectance is large. When $n_i$ is greater than 0.4, $R_{NL}$ is extremely large and at the same frequency $R_{NR}$ is extremely small, for example, $R_{NL}$ can be greater than 6, and at the same frequency, $R_{NR}$ can be smaller than 0.17. This phenomenon is termed unidirectional weak visibility^24^, which refers to the phenomenon wherein the reflectance from one side is extremely small and the reflectance from the other side is extremely large in forbidden gaps. This phenomenon can be also explained by applying the generalized conservation law to the following case $\sqrt{R_{NL}R_{NR}}=1$. A few similarities and differences are observed between this phenomenon and well-known unidirectional invisibility. In unidirectional invisibility, the reflectance from one side is zero and reflectance from the other side is nonzero. However, in our phenomenon, the reflectance from one side is extremely small but not zero, while the reflectance from the other side is extremely large. Moreover, in unidirectional invisibility, the transmittance is one, while in our phenomenon it is zero and occurs in the band gap. Imagine two people standing on both sides of a mirror. If this mirror supports the phenomenon of unidirectional invisibility, then one of these two people can see themselves in this mirror and the other person cannot. Moreover, one of them can see the other person, and the luminance obtained by the observer is the same as that obtained by the person being observed. However, if this mirror supports the phenomenon of unidirectional weak visibility by using our model, then one of these two people can see themselves more clearly in this mirror, and the other people can see themselves with weak luminance. Moreover, one of them cannot see the other person.

In addition to this phenomenon, in conduction bands, the corresponding Bloch wave vector *K* in Eq. (7) is real, thus $U_N={\sin NK\Lambda }/{\sin K\Lambda }$ is also real. Near exceptional points, *bc* becomes negative for $u^2<v^2$, which cannot be realized in Hermitian systems, and ${{\sin }^2 NK\Lambda }/{{{\sin }^2 K\Lambda }}$ approaches $\left| {1}/{bc}\right|$; thus, the transmittance $T_N$ and reflectances $R_{NL}$ and $R_{NR}$ can arrive at values greater than 1 simultaneously, which is shown in Eq. (7). For example, when $n_i=0.2$ in Fig. 4d and e, at the reduced frequency 0.249, $T_N$ reaches 5.2, and $R_{NL}$ and $R_{NR}$ reach 5.7 and 3.1 simultaneously. However, in other conduction bands and all forbidden gaps, $T_N$, $R_{NL}$, and $R_{NR}$ cannot reach large values simultaneously. This is because, in these regions, *bc* is greater than zero; thus, $T_N$ cannot be greater than one, which is shown in Eq. (7).

In previous studies^3,7,11^, researchers have investigated scattering properties by analysing the eigenequation of the scattering matrix. The scattering matrix of our model is given by $$S=\left( \begin{matrix} r_{NL} &{} t_N \\ t_N &{} r_{NR} \end{matrix}\right) $$, and the corresponding eigenvalues $s_{N1,N2}$ and the ratios $v_{N1,N2}$ of the amplitudes of two eigenstates can be derived:

8a $$\begin{aligned} s_{N1,N2}= & {} \frac{i \left[ (b+c)U_N \pm \sqrt{(b-c)^2U_N^2-4}\right] }{2 \left( aU_N-U_{N-1}\right) }, \end{aligned}$$

8b $$\begin{aligned} v_{N1,N2}= & {} \frac{i \left[ (c-b)U_N \pm \sqrt{(b-c)^2U_N^2-4}\right] }{2}. \end{aligned}$$

These two eigenvalues satisfy $\left| s_{N1} s_{N2} \right| =1$. When $(b-c)^2U_N^2-4<0$, $\left| s_{N1} \right| =\left| s_{N2} \right| =1$, that means energy is conserved in this case for an incident eigenstate. When $(b-c)^2U_N^2-4>0$, $s_{N1,N2}=1/s_{N2,N1}^*$. Hence, one eigenstate exhibits amplification and the other dissipation, and the degree of this amplification or dissipation can be measured by the absolute value of the corresponding eigenvalue. In our model, Eq. (1) shows that $b-c={2n_rn_i}\left[ {\cos \left( 2k_rd\right) }-{\cosh \left( 2k_id\right) }\right] /\left( n^2_r+n^2_i\right)$; thus, for Hermitian systems, $(b-c)^2U_N^2-4<0$ is always fulfilled.

In unidirectional weak visibility, the scattering matrix is diagonal, the eigenstates are the incident waves from left and right sides, and the corresponding eigenvalues are the corresponding reflections $r_{NL}$ and $r_{NR}$. The scattering of one eigenstate exhibits amplification, while the other eigenstate dissipation. Thus, under zero transmittance, the reflectance from one side can be extremely large, and simultaneously, the reflectance from the other side can be extremely small.

**Figure 5:**
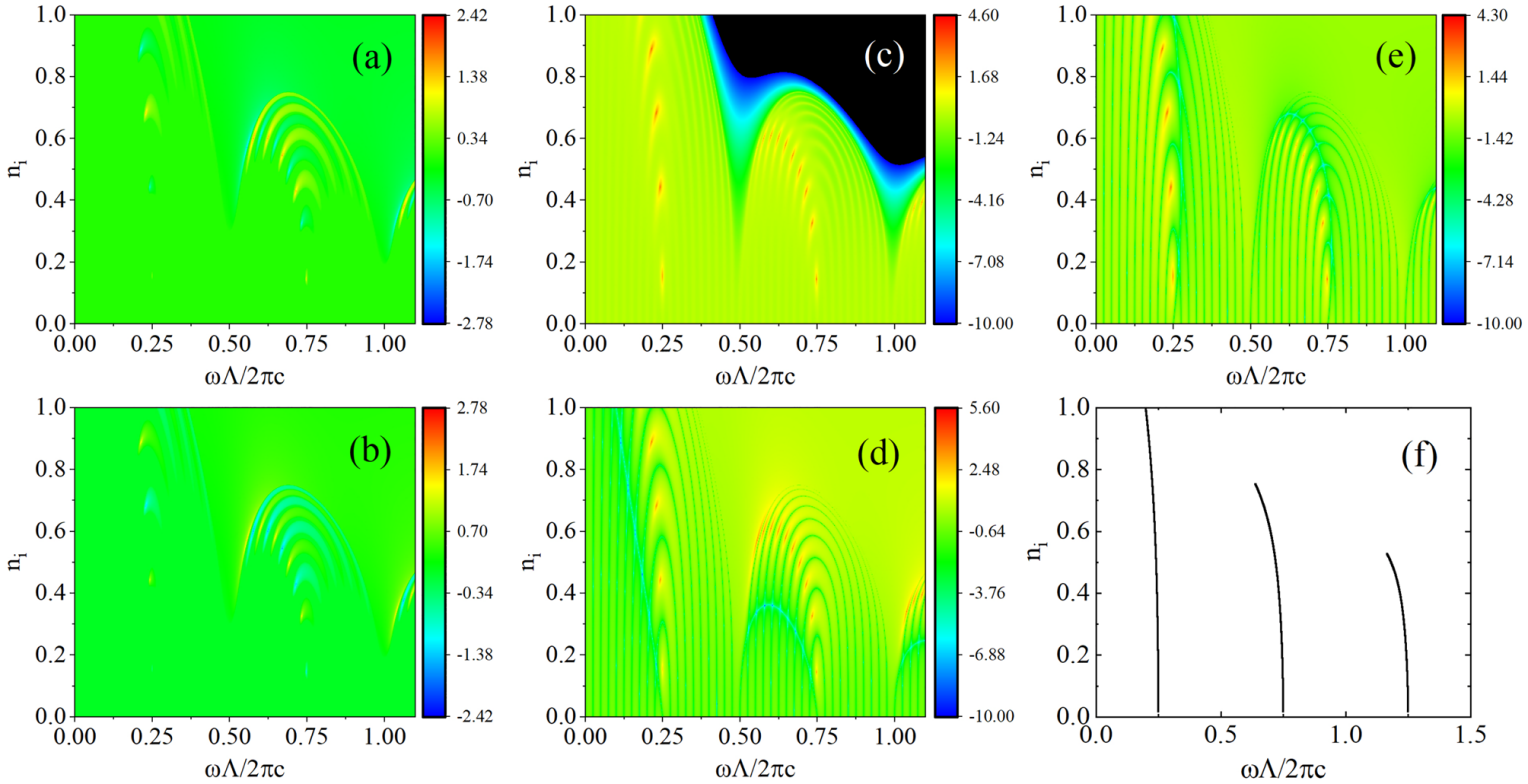
Eigenvalues $\text {log}_{10} |s_{N1}|$ (**a**) and $\text {log}_{10} |s_{N2}|$ (**b**) of the scattering matrix of SOPTPC as functions of the reduced frequency $\omega \Lambda /{2\pi c_0}$ and imaginary part of refractive index $n_i$. (**c**) $\text {log}_{10}(T_{N})$, (**d**) $\text {log}_{10}(R_{NL})$, and (**e**) $\text {log}_{10}(R_{NR})$ in the space $(\omega \Lambda /{2\pi c_0}, n_i)$. (f) Trajectory of the exceptional points in the same space $(\omega \Lambda /{2\pi c_0}, n_i)$.

To comprehensively and thoroughly study the eigen-equation of the scattering matrix, we plot its two eigenvalues as functions of reduced frequency and imaginary part of the refractive index in Fig. 5a and b. We determine that the poles and zeros of the scattering matrix are distributed discretely, and they turn up alternately. We believe the values of eigenvalues shown in these figures are not 0 or $\infty$, because in our calculation, the number of the points we scan in the space $(\omega \Lambda /{2\pi c_0}, n_i)$ is finite. Thus, if we magnify some poles and zeros, the corresponding eigenvalues near the poles and zeros are increased and decreased, respectively. These phenomena conform to our theory; based on our calculation, at these positions $bcU_N^2+1=0$. The relation between the pole and zero of the scattering matrix and singular scattering can be clearly observed in Fig. 5c–e, where $\text {log}_{10}(T_{N})$, $\text {log}_{10}(R_{NL})$, and $\text {log}_{10}(R_{NR})$ in the same space $(\omega \Lambda /{2\pi c_0}, n_i)$ are depicted. At the pole and zero of the scattering matrix, singular scattering occurs. In addition, the black and blue regions in Fig. 5c are forbidden gaps with zero transmittance, wherein unidirectional weak visibility occurs, which also can be confirmed in Figs. 5d and e. Figure 5f shows the trajectories of exceptional points in the same space obtained from dispersion relation. Comparing Fig. 5c–e with Fig. 5f, the points of singular scatterings are distributed discretely close to the trajectories of the exceptional points. When the number of unit cells *N* is increased, more points of singular scatterings are observed. This phenomenon can be also elucidated by our theory—*b* and *c* are independent of *N*, and there are more points of $U_N^2$ along a trajectory satisfying $bcU_N^2+1=0$ for larger *N*, which can be understood from the function diagram of $U_N^2$. This means that more singular scatterings can be realized easily for more unit cells. Exceptional point is the coalescence point in general non-Hermitian system, where eigenstates become parallel with the same eigenvalue. Several novel properties near the exceptional point are disclosed and applied for the design of optical devices^28–30^. In the future, we intend to explore these properties and applications in the proposed model.

## Conclusion

Based on the band structure and eigen equation of a scattering matrix, we studied the scattering properties of SOPTPC. The analytic expressions of the transfer matrix of its unit cell were derived; furthermore, the analytic expressions of band structure and scattering properties were presented. These theoretical relations helped determine and elucidate the following phenomena: As non-Hermiticity is increased, exceptional point pairs appear from Brillouin boundaries at nonzero non-Hermiticity, then move to and disappear at the Brillouin center. Singular scattering, which is that transmittance and reflectances from both sides reach infinity simultaneously, emerges near exceptional points and is located at $bc U_N^2+1=0$; simultaneously, the pole and zero of the scattering matrix are reached. Unidirectional weak visibility in forbidden gaps is disclosed and analyzed by mathematical expressions; simultaneously, the incident waves from left and right sides are the eigenstates of the scattering matrix, and the corresponding reflections are the eigenvalues. We believe that our results are beneficial for the design of ${\mathscr {P}}{\mathscr {T}}$-symmetric photonic devices.

## Data Availability

Data underlying the results presented in this paper are not publicly available at this time but may be obtained from the calculation by authors.
